# Genomic signatures of population decline in the malaria mosquito *Anopheles gambiae*

**DOI:** 10.1186/s12936-016-1214-9

**Published:** 2016-03-24

**Authors:** Samantha M. O’Loughlin, Stephen M. Magesa, Charles Mbogo, Franklin Mosha, Janet Midega, Austin Burt

**Affiliations:** Department of Life Sciences, Imperial College London, Silwood Park, Ascot, SL5 7PY UK; NIMR Amani Research Centre, P.O. Box 81, Muheza, Tanzania; Global Health Division, RTI International, Dar es Salaam, Tanzania; Centre for Geographic Medicine Research Coast, Kenya Medical Research Institute, P.O. Box 428, Kilifi, Kenya; Malaria Public Health Department, Centre for Geographic Medicine, KEMRI-Wellcome Trust Research Programme, Kenyatta National Hospital Grounds, P.O. Box 43640-00100, Nairobi, Kenya; Kilimanjaro Christian Medical University College, Moshi, Tanzania; Department of Life Sciences, Imperial College London, South Kensington Campus, London, SW7 2AZ UK; Wellcome Trust Centre for Human Genetics, Oxford, OX3 7BN UK

**Keywords:** *Anopheles gambiae*, Kilifi, Linkage disequilibrium, Population recombination, Population control, Pest management

## Abstract

**Background:**

Population genomic features such as nucleotide diversity and linkage disequilibrium are expected to be strongly shaped by changes in population size, and might therefore be useful for monitoring the success of a control campaign. In the Kilifi district of Kenya, there has been a marked decline in the abundance of the malaria vector *Anopheles gambiae* subsequent to the rollout of insecticide-treated bed nets.

**Methods:**

To investigate whether this decline left a detectable population genomic signature, simulations were performed to compare the effect of population crashes on nucleotide diversity, Tajima’s D, and linkage disequilibrium (as measured by the population recombination parameter ρ). Linkage disequilibrium and ρ were estimated for *An. gambiae* from Kilifi, and compared them to values for *Anopheles arabiensis* and *Anopheles merus* at the same location, and for *An. gambiae* in a location 200 km from Kilifi.

**Results:**

In the first simulations ρ changed more rapidly after a population crash than the other statistics, and therefore is a more sensitive indicator of recent population decline. In the empirical data, linkage disequilibrium extends 100–1000 times further, and ρ is 100–1000 times smaller, for the Kilifi population of *An. gambiae* than for any of the other populations. There were also significant runs of homozygosity in many of the individual *An. gambiae* mosquitoes from Kilifi.

**Conclusions:**

These results support the hypothesis that the recent decline in *An. gambiae* was driven by the rollout of bed nets. Measuring population genomic parameters in a small sample of individuals before, during and after vector or pest control may be a valuable method of tracking the effectiveness of interventions.

**Electronic supplementary material:**

The online version of this article (doi:10.1186/s12936-016-1214-9) contains supplementary material, which is available to authorized users.

## Background

Many population genomic parameters depend upon population size. These include genetic diversity (θ or π), linkage disequilibrium, the population recombination parameter ρ, and runs of homozygosity [1–4]. Changes in population size can also lead to transient changes in the allele frequency spectrum and statistics based on it (e.g. Tajima’s D; [5, 6]). Thus population genomic data contain information on the past demographic history and might, therefore, show the effects of efforts to suppress the population, as has been observed in the *Plasmodium* genome after malaria control [7, 8].

In many parts of Africa there have been concerted efforts to control malaria transmission by controlling the mosquito vector using insecticide treated bed nets (ITNs) and indoor residual spraying (IRS). In some places these efforts have been successful, resulting in substantial reductions in the numbers of *Anopheles gambiae* (the most important vector species in sub-Saharan Africa) and in malaria transmission [9, 10]. *An. gambiae* is particularly susceptible to ITNs and IRS because of its propensity to bite and rest indoors. Other vectors may be less susceptible to these control methods, including the sibling species *Anopheles arabiensis*, which is able to bite earlier and outdoors due to increased resistance to desiccation [10, 11].

One place where there has been a particularly striking reduction in *An. gambiae* abundance is in the Kilifi district of coastal Kenya: entomological surveys have revealed an overall reduction in density of *An. gambiaes.l*. and *Anopheles funestus*, accompanied by a shift in the proportions of different species, with *An. arabiensis* and *Anopheles merus* replacing *An. gambiae s.s.* and *An. funestus* as the major vectors [12]. The authors attribute this shift in species composition to the widespread distribution and use of ITNs from 2006 onwards, although they do not rule out land-use change and improvements in house construction as contributing factors.

A previous study by O’Loughlin et al. [13] reported a RADseq analysis of the *An. gambiaes.l.* species complex from three locations approx. 200 km apart in East Africa: Moshi, Muheza, and Kilifi. Although mosquito control is also being carried out in Muheza and Moshi, at the time of sampling there had been no reported decline in mosquito numbers. In this study it was found that genetic diversity in *An. gambiae* s.s. was slightly but significantly lower in Kilifi than in Muheza (~5 % lower π and ~15 % lower θ_W_ averaged across all chromosomes). Diversity did not differ among the three *An. arabiensis* populations. The study also found that *An. gambiae* in Kilifi was the only population with a positive value for Tajima’s D, reflecting a deficit in low frequency polymorphisms, consistent with a recent decline in population size [14]. Modelling of the allele frequency spectra showed that *An. arabiensis* and *An. merus* fitted a simple model of modest population expansion, whereas the *An. gambiae* populations showed a more complex history of past population expansion followed by population decline. In the case of *An. gambiae* from Kilifi, the present population size was inferred to be smaller than the historical, pre-expansion size.

These results appear to be consistent with a recent population reduction for *An. gambiae* in Kilifi, perhaps due to control efforts [13]. To investigate this hypothesis more closely, here the analysis is expanded to consider linkage disequilibrium and the population recombination parameter ρ. RADseq data consists of a small fraction of the genome so is not suitable for some linkage-based methods of inferring population history such PSMC and MSMC [15, 16]. However the number of SNPs and their location throughout the genome make it ideal for calculating ρ. In the standard neutral model at equilibrium ρ has an expected value (or is defined as) 4N_e_r, where N_e_ is the effective population size and r is the recombination rate per base per generation. ρ is inversely related to the levels of linkage disequilibrium in a sample. It has previously been observed, both empirically and by simulation, that ρ is strongly affected by non-equilibrium demographics and selection [3, 17]. Although it is well established that ρ decreases after population bottlenecks followed by recovery (e.g. [3, 17, 18]), these studies did not explore the effect of very recent population declines without recovery, such as after successful vector control. Therefore, in this study, simulations are used to study the time-scale over which the different population genomic parameters are expected to change in response to reductions in population size.

## Methods

### Simulations: genomic signatures of successful control

To investigate the expected effect of a recent population crash on ρ, θ_W_, π and Tajima’s D, sequences were simulated under different demographic scenarios using Hudson’s ms [19]. A sample size of n = 26 was used (equivalent to 13 diploid individuals), chosen to match the sample size that were analysed with RADseq, and simulated sequences of 50 kb in length. Populations were simulated with an ancestral size of 2 million (the estimated long term N_e_ for *An. gambiae* population from [13]), a mutation rate of 1.1 × 10^−9^ per generation (estimated from divergence of *Drosophila* lineages [20] and assuming ten generations per year), and a ρ of 10 times present θ, which is the neutral expectation of ρ/θ calculated using the recombination rate for chromosome 3L [21] and is within the range of values seen at selective and demographic neutrality in *Drosophila* populations [22, 23]. Throughout the simulations parameters for the 3L chromosome arm were used, because 2L and 2R contain polymorphic inversions in *An. gambiae* and similarly 2R and 3R in *An. arabiensis*. Population crashes were simulated in which the population size after the crash was 10^−2^, 10^−3^, 10^−4^ or 10^−5^ of the ancestral population, and occurred 10, 10^2^, 10^3^ or 10^4^ generations in the past. Ms commands are given in Additional file 1.

### Empirical data from East Africa

Details of mosquito collections, RADseq analysis and SNP genotyping are given in [13]. Briefly, *An. gambiae*, *An. arabiensis* and *An. merus* were collected in Kilifi (Kenya), *An. gambiae* and *An. arabiensis* from Muheza (Tanzania, about 200 km south of Kilifi), and *An. arabiensis* from Moshi (Tanzania, about 200 km south-west of Kilifi). *An.gambiae* and *An.arabiensis* were collected in May–July 2010; *An.merus* in Oct 2009 and May 2010. Villages were of similar size, and house construction was the same in all locations. Genomic DNA was used for RADseq analysis with the SbfI enzyme (Floragenex, Oregon). RADseq reads were aligned to the *An. gambiae* PEST reference genome using BWA v0.5.9 [24] and SNPs were called in SAMtools v0.1.18 [25]. Summary genetic diversity statistics for each species and sampling location were reported in [13]. Metrics of data used are given in Table 1.

**Table 1:** Metrics of data sets containing variant and invariant sites

Species	No. indivs.	No. of tag locations	No. bp	Mean coverage	Mean dist between RADseq tags (kb)	No. of SNPs^a^	Mean (Max) SNPs per tag
*An. gambiae*	24	2033	172,655	88	131	6387 (6437)	3.14 (17)
*An. arabiensis*	36	2049	129,315	106	130	5137 (5195)	2.51 (23)
*An. merus*	12	2280	253,043	134	115	4135 (4161)	1.81 (13)

^a^Number in brackets shows number of SNPs in variant only data sets. Bayesian variant calling resulted in slightly different numbers of SNPs in variant only vs genotype data sets

r^2^ was calculated for SNPs in Haploview v4.2. The expected value of r^2^ is a function of the recombination rate and effective population size (N_e_), but is also affected by sample size [2], so the r^2^ was adjusted for the sample size component by subtracting 1/n (see Eq. 1 in [2], n = number of diploid individuals). ρ was estimated for SNPs using the composite likelihood method implemented in LDhat [26]. Under the standard neutral model ρ/θ ≈ r/μ, so the expectation of ρ/θ at neutrality can be calculated using estimated recombination rates for each *An.gambiae* chromosome arm taken from [21] and [27], and a mutation rate (μ) of 1.1 × 10^−9^ per generation [20]. To investigate what combinations of severity and timing of decline are consistent with observed ρ values, further simulations were carried out, simulating alternative demographic scenarios using ms [19]. As before, 50 kb segments were simulated, with n = 26, μ = 1.1 × 10^−9^ and ρ set at 10 × current θ, but this time the ancestral population size was adjusted such that final value of π was as close as possible to that observed in the Kilifi *An. gambiae* population (π = 0.0081). To do this the standard recursion equation for π was used (e.g. [28]).

$$\pi_{t} = \pi_{t - 1} \left( {1 - \frac{1}{{2 N_{2} }}} \right) + 2\mu \left( {1 - \pi_{t - 1} } \right)$$

with the initial value $$\begin{aligned} \hfill \\ {{\pi_{0} = 4N_{ 1} \mu } \mathord{\left/ {\vphantom {{\pi_{0} = 4N_{ 1} \mu } {\left( { 1 + 4N_{ 1} \mu } \right)}}} \right. \kern-0pt} {\left( { 1 + 4N_{ 1} \mu } \right)}} \hfill \\ \end{aligned}$$ being the equilibrium nucleotide diversity before the crash, π_*t*_ the diversity *t* generations after the crash, *N*_*1*_ and *N*_*2*_ the effective population sizes before and after the crash, respectively, and *m* the mutation rate. For particular values of the magnitude of population reduction (*N*_*2*_*/N*_*1*_ = 10^−2^, 10^−3^, 10^−4^, 10^−5^) and for particular values of time since the crash (*t* = 10, 10^2^, 10^3^, and 10^4^ generations ago) these equations were used to find the starting value of π_*0*_ that would give a π_*t*_ close to the observed value (0.0081). These values of *N*_*1*_, *N*_*2*_, and *t* were then used to generate sequences using ms [19]. 10 simulations of each scenario were performed. The ms output was converted to diploid format for estimating ρ in LDhat (to simulate 13 diploid samples). A regression analysis was then performed, of ρ as the dependent variable and crash size and generations in the past as independent variables.

Another potential sign of population crash is extended runs of homozygosity caused by mating between related individuals [4, 29]. To look for runs of homozygosity, heterozygosity at every site was plotted across the genome for each mosquito individually.

## Results

### Simulations: genomic signatures of successful control

**Fig. 1:**
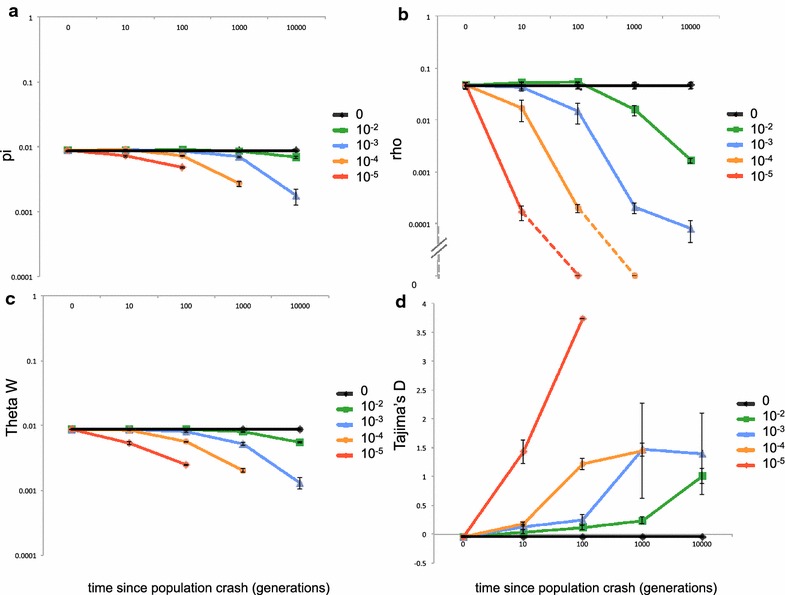
Simulations to show the genomic consequences of population crashes of different strengths and timing. **a** π; **b** ρ; **c** θ_W_; **d** Tajima’s D. Each* plot* shows simulations of different size population crashes at 10, 100, 1000 and 10,000 generations before present (*x-axis*), with post-crash population sizes of 10^−2^, 10^−3^, 10^−4^ and 10^−5^ (*lines*). **a**–**c** *y-axis* is log10 scale. *Dashed lines* indicate two ρ values that were zero. *Error bars*= ± one standard error. Based on five simulations per scenario except decline of 10^−5^ 1000 gens ago where only two simulations resulted in sufficient segregating sites for calculating statistics

The population genetic parameters from the simulated data sets are shown in Fig. 1. Strong population decline a long time in the past gave too few segregating sites for analyses (due to a long-term low N_e_), so some curves are truncated. As expected, population declines lead to reductions in π, θ_W_ and ρ, and increases in Tajima’s D, with larger reductions in population size producing larger and more rapid effects on the population genomic statistics. (Simulating reductions even further in the past than 10,000 generations resulted in Tajimas’s D returning towards zero (results not shown); this reflects the fact that the population has remained small but stable for a long enough time equilibrium to be restored.) Importantly, for all scenarios, ρ responded faster and/or by a larger relative amount than the other three statistics, suggesting it can be a more sensitive measure of population control.

### Empirical data from East Africa

#### Linkage disequilibrium

**Fig. 2:**
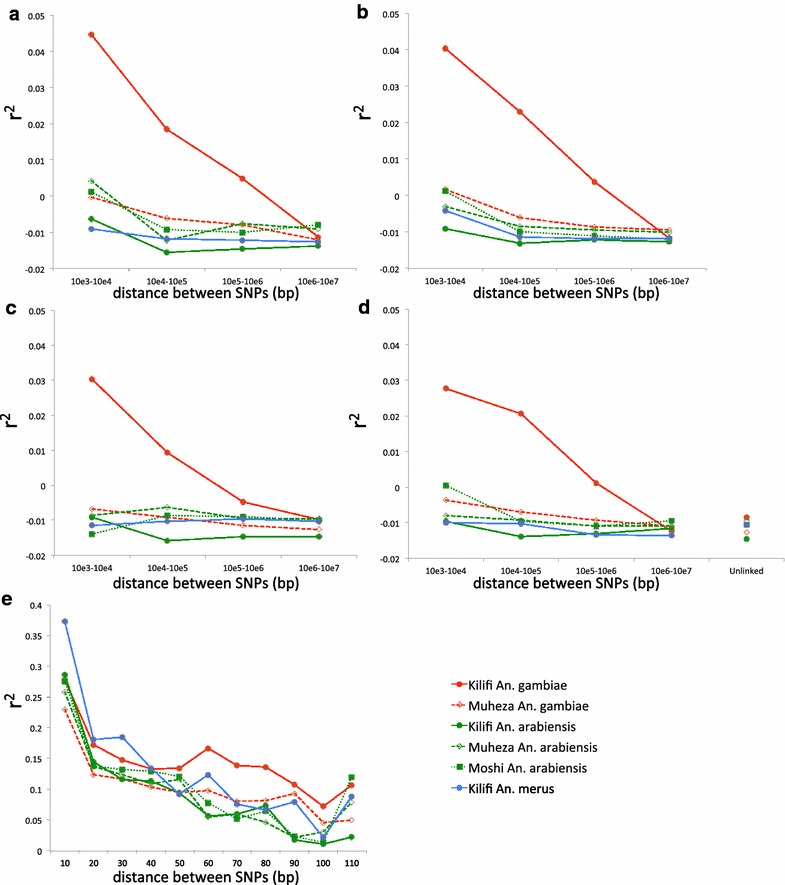
Sample size adjusted r^2^ values for autosomes by genetic distance. **a**–**d** The r^2^ values were averaged in log_10_ bins for each autosome arm separately. **a** 2L; **b** 2R; **c** 3L; **d** 3R. **d** also shows the average r^2^ for unlinked loci on separate chromosomes (specifically between sites on 2L and 3L). **e** The r^2^ values were averaged across bins of 10 bp from 0 to 110 and averaged for all autosomes. SNPs within the 2La, 2Rb and 3Ra inversions were removed before r^2^ was calculated

To compare linkage disequilibrium among populations and species, the analysis was restricted to the autosomes, and because inversions greatly increase linkage disequilibrium (see Additional file 2), SNPs in the segregating inversions in the samples (2La, 2Rb and 3Ra) were removed from all calculations in all species whether the inversion was segregating or not, to ensure only collinear regions of the genome were used. For each chromosome arm, pairwise linkage disequilibrium (r^2^) between polymorphic sites in different RADseq tag locations was calculated and averaged in bins from 10^3^ to 10^7^ bp (Fig. 2). For all four chromosome arms, linkage disequilibrium is substantially higher in the Kilifi population of *An. gambiae* than in any of the other populations. In these other populations linkage disequilibrium is at background levels (i.e., equal to that between polymorphisms on different chromosomes – Fig. 2d) at distances of 1–10 kb or greater, whereas for *An. gambiae* from Kilifi this only occurs at distances greater than 1 Mb. Linkage disequilibrium between polymorphisms in the same RADseq tag location (length 110 bp) were also analysed, though there are many fewer such pairs of polymorphisms and so data was combined across the chromosomal arms (Fig. 2e). There is some tendency for linkage disequilibrium to be higher in Kilifi *An. gambiae* than in other populations, particularly for markers separated by 50–110 bp, though the difference is not as dramatic.

#### Population recombination parameter ρ

**Fig. 3:**
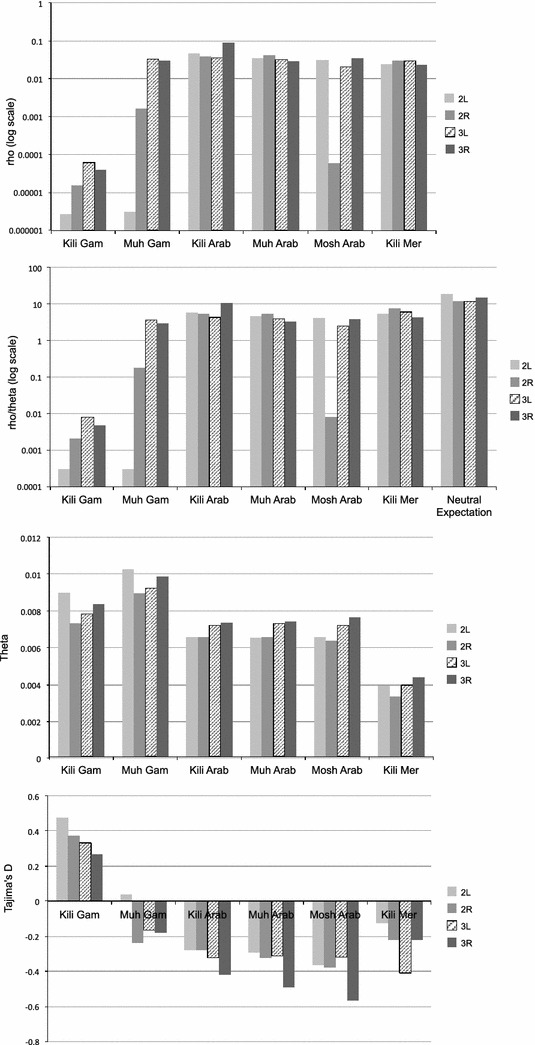
Empirical data: θ_W_, Tajima’s D, ρ and ρ/θ_W_ for each species, population and autosome. ρ and θ_W_ are per base. Note the log scale on the *y-axis* for ρ and ρ/θ_W_. Inversion-less chromosome 3L is hatched for emphasis. Tajima’s D and θ_W_ from [13]

In Fig. 3, estimates of ρ and ρ/θ_W_ for each species, sampling location and chromosome arm are shown. For ease of comparison, θ_W_ and Tajima’s D from [13] are also given. Setting aside the population of *An. gambiae* from Kilifi, one can see that ρ is relatively consistent across species, populations and chromosome arms, with the exception of 2L for *An. gambiae* in Muheza and 2R for *An. arabiensis* in Moshi, for both of which ρ is much reduced (Fig. 3a). Both these samples have segregating inversions in the relevant arm which presumably accounts for the reduction, though it is not clear why the effect is not seen for 2R in the other populations of *An. arabiensis.* ρ is also somewhat reduced in 2R for *An. gambiae* in Muheza; there is an inversion segregating on this arm in East African *An. gambiae*, which, even if not segregating in these samples, would reduce recombination and could account for the lower value. Apart from these exceptions, the ratio ρ/θ_W_ is close to its neutral expectation (=r/u), though consistently somewhat lower (Fig. 3b), perhaps because r has been over-estimated, or u under-estimated, or because of population structure [30].

The results from the Kilifi population of *An. gambiae* are markedly different, with ρ low in all arms. Comparison with the Muheza population demonstrates a statistically significant difference (paired t test, t = 2.39, p = 0.04). The reduction in ρ in Kilifi vs Muheza is between 11 % (2L) and 99.9 % (3R), with an average across chromosomes of 82 %. This difference is substantially greater than the reductions seen in diversity measures π and θ_W_. As a result, the ratio ρ/θ_W_ for *An. gambiae* from Kilifi is more than 1000-fold lower then neutral expectations. The comparison among populations and species is perhaps clearest for chromosome arm 3L, which does not have segregating inversions in any of these three mosquito species (hatched bars in Fig. 3).

#### Estimating the timing and severity of population crash from ρ

**Fig. 4:**
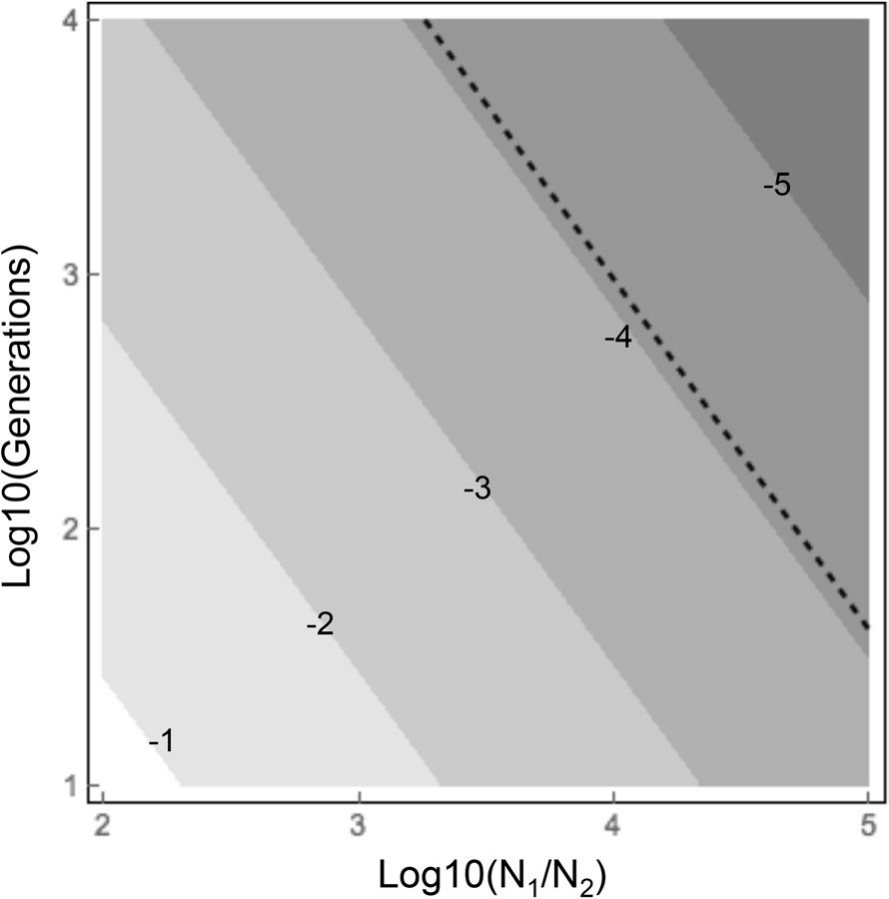
*Contour plot *from regression model. Contours show predicted values of log (ρ + 1/50,000). *Dotted line* shows the estimated ρ value from Kilifi samples

To investigate the combination of timing and extent of population decline consistent with the observed value of ρ for Kilifi *An. gambiae*, population crashes of varying magnitude and at varying times in the past were simulated, in each case keeping the final value of π as close as possible to that currently observed in this population (π = 0.0081). Simulations of a population decline of 10^−5^ more than 100 generations in the past gave highly variable results and so were excluded from further analysis. For the remaining simulations a linear regression model was fitted using Fit Model in JMP v12.0.1 (SAS Institute Inc., Cary, NC). The best fitting model was

$${\text{Log}}10\,\left( \uprho \right) = 1.977 - 0.983 \times {\text{Log}}10\,\left( {{{{\text{N}}_{1} } \mathord{\left/ {\vphantom {{{\text{N}}_{1} } {{\text{N}}_{2} }}} \right. \kern-\nulldelimiterspace} {{\text{N}}_{2} }}} \right) - 0.717 \times {\text{Log}}10\,\left( {{\text{Generations}}} \right)$$

where ρ = ρ per base + 1/50,000 (to remove zero values), Generations = generations since population crash, N_1_ = pre-crash population size, N_2_ = post-crash population size. The model r^2^ was 0.884. A contour plot of the model is shown in Fig. 4, which also shows the contour line corresponding to the ρ value from Kilifi gambiae 3L [with the same transformation as the simulations; Log10 (ρ per base + 1/50,000)]. The ρ value for Kilifi is compatible with a range of population crash sizes from 5 × 10^−4^ to 4 × 10^−6^ corresponding to times in the past from 10,000 generations ago to ten generations ago. The first distribution of bed nets in Kilifi occurred 17 years before the mosquitoes used in this study were collected, which equates to ~170 generations. According to the model, if the crash occurred between 10 and 170 generations ago, this would correspond to a large crash of between 3 × 10^−5^ (at 170 generations ago) and 4 × 10^−6^ (at 10 generations ago) of the ancestral population. In terms of a reduction in mosquito numbers, a reduction of 3 × 10^−5^ would mean that a starting population of one million mosquitoes per square km would be reduced to just 30 mosquitoes per square km.

#### Runs of homozygosity

**Fig. 5:**
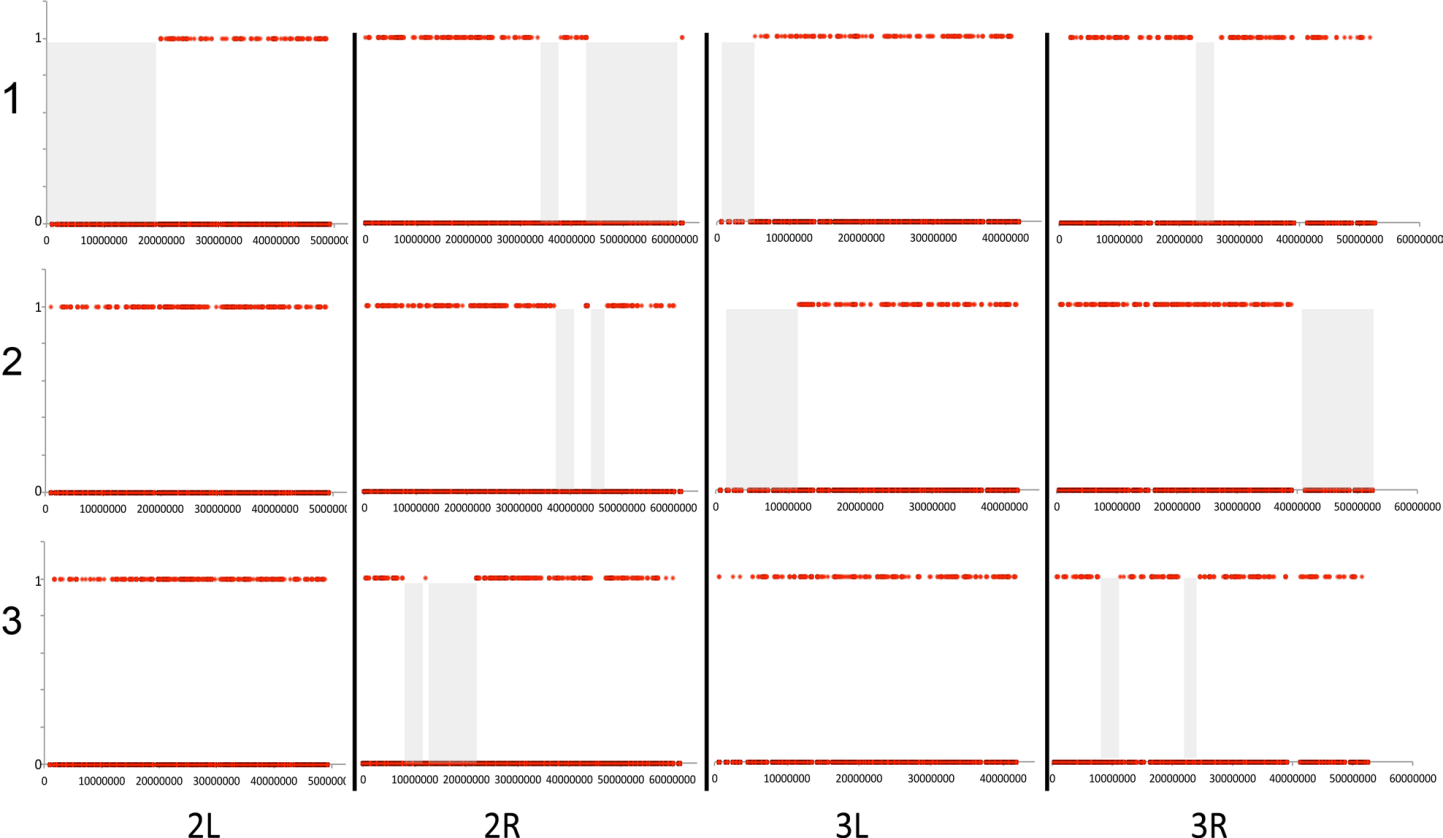
*Plots* of homozygosity for three Kilifi *An. gambiae* individuals. *Shaded areas* show runs of over 4.5 Mb where only homozygous sites are present

At least one long run of homozygosity (>4.5 Mb) was observed in 7 of the 13 Kilifi *An. gambiae* individuals, despite having RADseq data instead of full genome sequences. Examples of these are shown in Fig. 5. The runs of homozygosity in different individuals are in different genomic locations, and so cannot be attributed to a selective sweep. *Anopheles gambiae* from Muheza and all other species and populations do not show any such runs of homozygosity.

## Discussion

Vector control is an important tool in the fight against malaria and other vector borne diseases, but as yet measuring the entomological impact of these methods has been largely anecdotal or ad-hoc, with even large shifts in abundance or species proportions being difficult to quantify. This is because monitoring changes in mosquito numbers is not an easy activity; entomological surveying is labour-intensive and expensive, and prone to variation caused by seasonal fluctuations, different collection methods and degree of collection effort (e.g. [31, 32]). One alternative for detecting changes in population size may be monitoring the genome.

In the Kilifi district of Kenya, ITNs were first introduced in a randomized trial in 1993, when their effectiveness in reducing malaria incidence in children was proven [33]. Since then, ITN coverage increased gradually until a large-scale distribution program in 2006 resulted in coverage rising to 67 % [34]. Over a similar time period, entomological surveys in the Kilifi region have detected a decline in vector density (from 1990 to 2010), with an accompanying change in *An.gambiaes.l.* species composition [12]. *Anopheles gambiae* declined from 79 % of *An. gambiaes.l.* in 1997–1998 to an undetectable level in 2007–2008. The dominant *An.gambiae s.l.* species in 2007–2008 was *An. arabiensis* (93 %), with *An.merus* contributing 5 %.

This change in mosquito abundance makes it a good model system to test whether the effect of population decline can be detected in the genome. Previously it has been shown that *An. gambiae* from Kilifi have lower π and θ and higher Tajima’s D compared to those from Muheza, where there has been no reported change in mosquito abundance [13]. *Anopheles arabiensis* from the same locations did not show any differences. These results were consistent with the observed recent changes in abundance.

Here, simulations are presented showing that analysis of linkage disequilibrium and ρ can be a more sensitive test for population decline, as large signals are seen more quickly. Therefore, these statistics were compared among populations and species, and indeed linkage disequilibrium extends 100–1000 times further in Kilifi than in comparator populations, and ρ is 100–1000 times lower. Population genetic simulations indicate that the observed ρ value implies that the sampled population is no more than a 5 × 10^−4^ (1/2000th) of the ancestral population. The observed values would also be consistent with an even greater, recent population decline. The unusual runs of homozygosity in Kilifi *An. gambiae* also suggest that a recent and severe population crash has occurred, which is resulting in signs of inbreeding in some individuals.

One weakness of the simulations is that they do not take into account spatial structure, which is known to effect the extent of linkage disequilibrium, especially when all the samples are from a single sub-population in a large “stepping-stone” array [3, 30]. In general, there is little differentiation among these East African populations of the same species [13], but since the extent of differentiation is determined by the product of the migration rate and the population size, there may be some interaction between a population crash and population structure affecting linkage disequilibrium and ρ. The inferences would also be improved by analysing a time series of samples from before and after the population crash, but unfortunately pre-control samples were not available for analysis.

The fact that a similar pattern of increased linkage disequilibrium and reduced ρ was not observed in *An. arabiensis* and *An.merus* collected from Kilifi at the same time as the *An. gambiae* samples suggests that these species have not been affected by the same population decline, and is consistent with the entomological observations of a shift in species composition [12]. This difference among species supports the hypothesis that the population decline has largely been due to the use of ITNs, as these are expected to have a larger impact on the highly anthropophilic and indoor-biting *An. gambiae*, compared with partially zoophilic and outdoor biting species such as *An. arabiensis* and *An. merus* [35].

The results of the simulations and observed data from Kilifi show promise for the prospective monitoring of vector control efforts. Mosquitoes have ~10 generations per year, so measurements of ρ, θ, π and Tajima’s D taken pre-intervention and at one-yearly intervals should detect whether control is succeeding. Whole-genome sequencing is not necessary for measuring ρ. The simulated data suggests it is possible to get reliable estimates of ρ from as few as 300 segregating sites, so a medium-throughput SNP genotyping platform such as RADseq or Golden-Gate assay would be sufficient for monitoring. For *Anophele*s species it is important to use SNPs that are not on chromosome arms containing segregating inversions. The sample here of just 11–13 mosquitoes per population was sufficient to distinguish clear differences in ρ between populations and chromosome arms.

## Conclusions

Observations of genomic diversity and linkage disequilibrium in a small sample of just 13 mosquitoes provide compelling evidence that *An. gambiae* in Kilifi has undergone a recent population crash. In practical terms, this means that regular monitoring of a small number of genomes could allow rapid detection of whether a control intervention is succeeding. In nature, there may be complicating factors such as seasonal variation and immigration from non-treated areas, but the results presented here from Kilifi suggest that a severe population crash will be detectable despite these factors. Given the practical difficulties of measuring mosquito abundance by direct surveying, genotyping a small number of mosquitoes could be an attractive alternative for assessing the entomological impact of vector control.

Data available in Dryad Digital Repository: doi:10.5061/dryad.hm6tt.

## Additional files

10.1186/s12936-016-1214-9 Ms commands used for simulations.
10.1186/s12936-016-1214-9 Sample size adjusted LD decay curves for *An.* *gambiae* and *An. arabiensis* (collinear regions only, without 2La, 2Rb and 3Ra SNPs) and for *An.* *gambiae* 2La, *An. arabiensis* 2Rb and *An. arabiensis* 3Ra separately.
