# Supplementary material for: Genomic signatures of population decline in the malaria mosquito Anopheles gambiae
Source: Malar J. 2016 Mar 24;15:182. doi: 10.1186/s12936-016-1214-9 (PMC4806450; doi:10.1186/s12936-016-1214-9)
Supplement: Supplementary file 1 — 10.1186/s12936-016-1214-9 Ms commands used for simulations. [file 12936_2016_1214_MOESM1_ESM.docx]

**Genomic Signatures Of Population Decline In The Malaria Mosquito *Anopheles Gambiae***

Supplementary methods.

**Simulations: Genomic Signatures Of Successful Control**

Ms commands:

Constant population:

ms 26 10 -t 440.00 -r 4,400.00 50000 > 2mill100BNSim1

1/100 decline

ms 26 10 -t 4.40 -eN 0.0001250 100 -r 44.00 50000 > 2mill100BNSim2

ms 26 10 -t 4.40 -eN 0.0012500 100 -r 44.00 50000 > 2mill100BNSim3

ms 26 10 -t 4.40 -eN 0.0125000 100 -r 44.00 50000 > 2mill100BNSim4

ms 26 10 -t 4.40 -eN 0.1250000 100 -r 44.00 50000 > 2mill100BNSim5

1/1000 decline

ms 26 10 -t 0.44 -eN 0.0012500 1,000 -r 4.40 50000 > 2mill1KBNSim2

ms 26 10 -t 0.44 -eN 0.0125000 1,000 -r 4.40 50000 > 2mill1KBNSim3

ms 26 10 -t 0.44 -eN 0.1250000 1,000 -r 4.40 50000 > 2mill1KBNSim4

ms 26 10 -t 0.44 -eN 1.2500000 1,000 -r 4.40 50000 > 2mill1KBNSim5

1/10000 decline

ms 26 10 -t 0.04 -eN 0.0125000 10,000 -r 0.44 50000 > 2mill10KBNSim2

ms 26 10 -t 0.04 -eN 0.1250000 10,000 -r 0.44 50000 > 2mill10KBNSim3

ms 26 10 -t 0.04 -eN 1.2500000 10,000 -r 0.44 50000 > 2mill10KBNSim4

ms 26 10 -t 0.04 -eN 12.5000000 10,000 -r 0.44 50000 > 2mill10KBNSim5

1/100000 decline

ms 26 10 -t 0.0044 -eN 0.1250000 100,000 -r 0.044 50000 > 2mill100KBNSim2

ms 26 10 -t 0.0044 -eN 1.2500000 100,000 -r 0.044 50000 > 2mill100KBNSim3

ms 26 10 –t 0.0044 -eN 12.5000000 100,000 -r 0.044 50000 > 2mill100KBNSim4

ms 26 10 -t 0.0044 -eN 125.0000000 100,000 -r 0.044 50000 > 2mill100KBNSim5

Empirical Data From East Africa

Simulations keeping final value of *π *was as close as possible to that observed in the Kilifi *An. gambiae* population (*π*=0.0081). Ms commands:

Constant population:

ms 26 10 -t 409.20 -r 4092 50000 > Kili13

1/100 decline

./ms 26 10 -t 4.09 -eN 0.0001344 100 -r 40.92 50000 > Kili7

./ms 26 10 -t 4.09 -eN 0.0013441 100 -r 40.92 50000 > Kili1

./ms 26 10 -t 4.20 -eN 0.0130890 100 -r 42.02 50000 > Kili4

./ms 26 10 -t 5.06 -eN 0.1086957 100 -r 50.60 50000 > Kili10

1/1000 decline

./ms 26 10 -t 0.41 -eN 0.0013441 1000 -r 4.09 50000 > Kili8

./ms 26 10 -t 0.42 -eN 0.0130890 1000 -r 4.20 50000 > Kili2

./ms 26 10 -t 0.51 -eN 0.1082251 1000 -r 5.08 50000 > Kili5

./ms 26 10 -t 1.11 -eN 0.4950495 1000 -r 11.11 50000 > Kili11

1/10000 decline

./ms 26 10 -t 0.04 -eN 0.0130890 10000 -r 0.42 50000 > Kili9

./ms 26 10 -t 0.05 -eN 0.1082251 10000 -r 0.51 50000 > Kili3

./ms 26 10 -t 0.11 -eN 0.4940711 10000 -r 1.11 50000 > Kili6

./ms 26 10 -t 0.47 -eN 1.1792453 10000 -r 4.66 50000 > Kili12

1/1000000 decline

./ms 26 10 -t 0.01 -eN 0.1082251 100000 -r 0.05 50000 > Kili14

./ms 26 10 -t 0.01 -eN 0.4930966 100000 -r 0.11 50000 > Kili15

./ms 26 10 -t 0.05 -eN 1.1792453 100000 -r 0.47 50000 > Kili16
