# Supplementary figures and images for: Genomic signatures of population decline in the malaria mosquito Anopheles gambiae
Source: Malar J. 2016 Mar 24;15:182. doi: 10.1186/s12936-016-1214-9 (PMC4806450; doi:10.1186/s12936-016-1214-9)

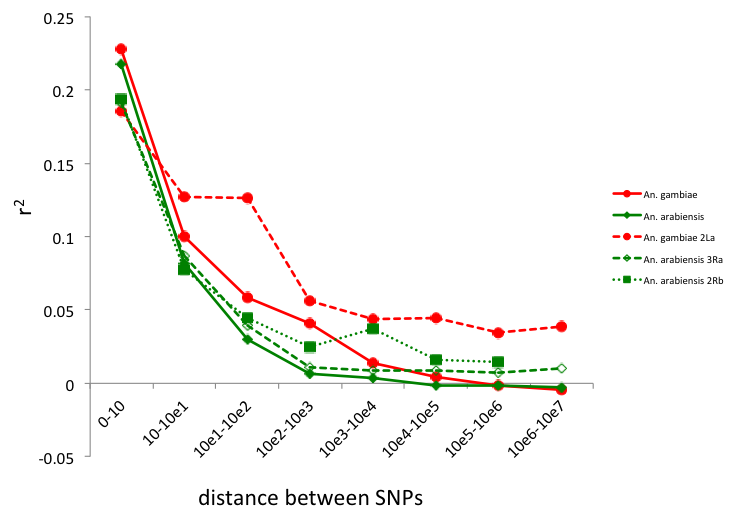

Supplement: Supplementary file 2 — 10.1186/s12936-016-1214-9 Sample size adjusted LD decay curves for An. gambiae and An. arabiensis (collinear regions only, without 2La, 2Rb and 3Ra SNPs) and for An. gambiae 2La, An. arabiensis 2Rb and An. arabiensis 3Ra separately. [file 12936_2016_1214_MOESM2_ESM.png]
